# A Cross-Sectional Study of Sports Food Consumption Patterns, Experiences, and Perceptions amongst Non-Athletes in Australia

**DOI:** 10.3390/nu16081101

**Published:** 2024-04-09

**Authors:** Celeste I. Chapple, Alissa J. Burnett, Julie L. Woods, Catherine G. Russell

**Affiliations:** School of Exercise and Nutrition Sciences, Institute for Physical Activity and Nutrition (IPAN), Deakin University, Geelong, VIC 3220, Australia

**Keywords:** sports foods, sports supplements, sports nutrition, food labelling, food choice, food choice behaviour

## Abstract

Sports foods are designed for athletes, yet their availability, type, and sales have increased over the past decade, likely driven by non-athlete use. This could lead to detrimental health outcomes via over/misuse or unwanted side effects. The aim of this study was to describe sports food consumption patterns and associated drivers, consumption reasons, perception of risks, and side effects experienced amongst non-athletes in Australia. In 2022, *n* = 307 non-athlete Australian adults (18–65 years) completed an online cross-sectional survey including closed-ended (consumption patterns, factors, and exercise participation) and open-ended questions (reasons for consumption, risk perception, and side effects experienced). Descriptive statistics (frequency and percent) described the sample. Ordinal logistic regression was used for univariate associations and a multivariate model was used to determine relationships between sports food consumption proxy and significant univariate associations. The themes were analysed via inductive thematic analysis using NVivo 14. Females consumed sports foods most frequently, 65% of participants consumed three or more sports foods, and participants with higher sports food consumption/frequency were less likely to perceive risks or experience side effects. The main reason for consumption was protein intake, digestion/stomach issues were the main perceived risks, and the main side effect was bloating. Despite understanding the risks and side effects, non-athlete consumers continue to use numerous sports foods, which appear to be influenced by sociodemographic factors and packaging labels. Tighter regulation of packaging-label information would ensure safer and more informed consumption.

## 1. Introduction

Protein powders/snacks/bars, carbohydrate powders/gels and other products such as beta alanine, L-carnitine, and high-caffeine foods such as pre-workout foods are specially formulated products. These foods contain specific amounts of macronutrients, micronutrients, and nutritive substances, not found in the same quantities as regular foods [1,2], and are not permitted to be sold in capsule or tablet form as dietary supplements nor be medications obtained through prescription. In Australia, these products are known as Formulated Supplementary Sports Foods (herein referred to as sports foods) [1] and are required to display warning labels stating the target consumer, conditions of use, and restrictions to daily consumption on their packaging [1,3]. Similar naming and regulations relating to compositional and packaging label requirements are used globally for these food types [4,5,6,7]. Sports foods are designed for athletes, who have increased nutrition requirements, not for the non-athlete population [1,8]. The consumption of sports foods by non-athletes poses risks of nutrient overconsumption [9] or the potential for unwanted side effects (e.g., caffeine overload), which can be further exacerbated by the stacking of products, defined as the consumption of two or more sports foods simultaneously to maximise results [10,11]. These foods can be purchased by non-athletes without the direction of a nutrition professional, contrary to their recommended/intended use, which can place consumers at risk of product overconsumption or misuse.

Over the past decade, a vast range of new products, available in mainstream retail settings (e.g., supermarkets) [12] has developed, alongside an increase of 152% in retail sales in Australia [13] and similar trends are seen throughout the world [14]. This suggests that sports foods are increasingly being sold in mainstream retail settings where they could be purchased by non-athletes [10,12]. Additionally, there has been growth in the media channels that sports foods are marketed through, such as social media and websites [12]. In order to understand why these foods are consumed by non-athletes, it is important to explore the factors that drive sports food choice in this population. This may enable food regulations that are fit for purpose, thereby promoting safer sports food consumption.

Food choice is complex, with varying levels and factors of influence with concomitant inter-relationships between factors. For the present study, the levels of influence for sports food choice were examined through the lens of the Social Ecological Model (SEM), which encompasses intrapersonal, interpersonal and sectors of influence [15]. Previous research examining the patterns and drivers of sports food consumption in Australia has suggested that intrapersonal factors such as gender, age, education level, income, physical activity level and perception of benefits (energy and muscle building), interpersonal factors such as recommendations from family, friends, work mates (peer groups), books, newspapers, and magazines, and sectors of influence such as packaging labels could influence choices [16]. The same research found that 71.9% of Australian consumers reportedly used packaging labels as the primary information source to determine the risks and benefits of consuming sports foods, suggesting that packaging labels are highly influential to sports food choices [16]. However, while this study provides important information about the general-population consumer, the data are largely outdated, having been conducted more than 10 years ago, and do not account for the expansion in sports food availability, the influence of packaging labels on the current consumer, the reasons why consumers are selecting these products, the perception of risks and side effects experienced, or the use of social media and websites in making sports food choices. These are all factors that are likely to be important influences on the decision to consume sports foods.

Consumers’ use of packaging information to determine sports food choice is concerning. A recent audit of these foods in Australian mainstream retailers found that over 50% of the available sports foods did not display the Food Standards Australia New Zealand (FSANZ) prescribed name or warning/advisory statement, and 33% had inaccurate nutrition information [12]. This lack of accurate information also calls into question the accuracy of nutrition content claims and general-level health claims, both of which are dependent on the stated nutrient information [12,17,18] and are important in consumer food-choice decisions [19,20,21,22]. Non-athletes, who may not have the benefits of dietetic guidance like athletes, could be consuming multiple sports food products using inaccurate packaging information and relying on nutrition claims that do not truly reflect the food item being considered [23,24], leading to overconsumption risks, potential side effects, and overall poor health outcomes. Therefore, it is important to examine the contemporary situation of the factors influencing consumption, including the role of packaging information, risk perception, and experiences of side effects.

The first aim of this study was to describe sports food consumption patterns (types, frequency, and amount), along with socioecological factors and drivers (e.g., gender, exercise participation, purchase location, source of recommendation, and packaging-information use) and their association with the consumption of sports foods among non-athletes in Australia. The second aim was to describe the reasons for consumption, the perception of risks and the experience of side effects amongst non-athlete Australian sports food consumers.

## 2. Materials and Methods

### 2.1. Design

The study was a cross-sectional, online self-report survey hosted on Qualtrics software version 05/2022 (Provo, UT, USA), among Australian adult, non-athlete consumers of sports food products, using closed and open-ended questions. A pilot survey was conducted prior to data collection and questions were amended based on the comments provided. The survey assessed sports food consumption patterns, reasons for consumption, perceived risks, side effects experienced, physical activity (exercise and incidental), purchasing, packaging use, and source by which sports foods were recommended to participants. The questions related to consumption, exercise participation, perceptions, purchasing, and recommendations were based off a Food Standards Australia New Zealand survey [16]. Sports food types were selected based on products identified from an Australian retail audit of sports foods [12] and included only those foods classified as Formulated Supplementary Sports Foods in Australia. Ethical approval was granted on the 5th May 2022 by Deakin University Human Ethics Advisory Group (HEAG-H 3_2022).

### 2.2. Participants, Inclusion/Exclusion Criteria

A convenience sample was recruited via paid advertisements on Facebook, Instagram, and through personal channels on LinkedIn and X (formally Twitter) between May and August 2022. Eligible participants were adults aged 18–65 years currently living in Australia who had consumed protein dominant, carbohydrate dominant, or other sports foods in the past 3 months.

**Figure 1:**
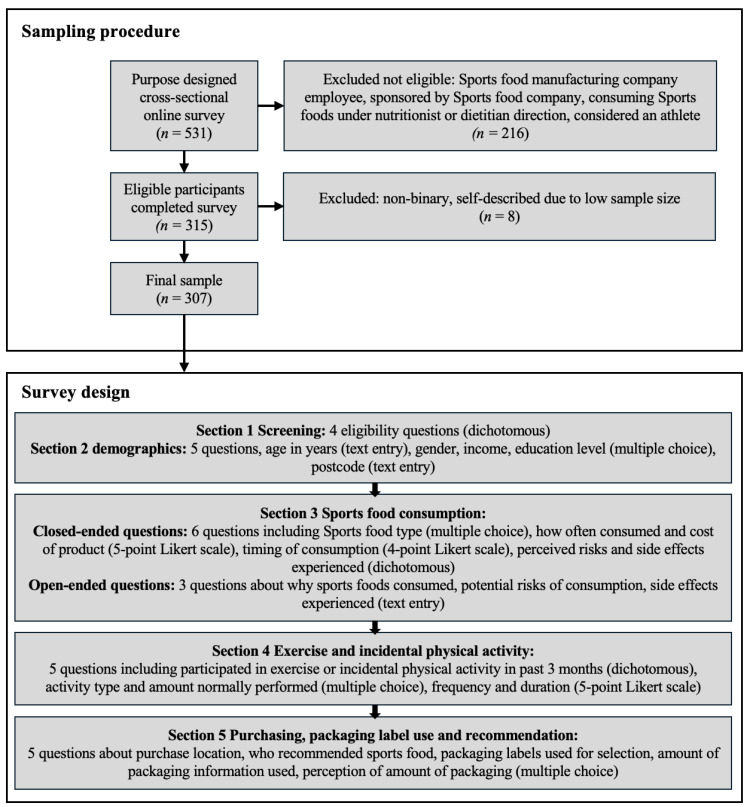
Participant sampling procedure and survey design.

Participants were excluded if they were employees of companies manufacturing sports foods, sponsored by a sports food company, or consuming sports foods under the direction of a dietitian/nutritionist. Participants were also excluded if they were classified as an athlete. Following Araujo and Scharhag [25], participants were classified as an athlete if they answered yes to all four criteria: participating in sport to improve performance/results; participating in sporting competitions; formally registered with a sports federation at any level; and sport is the major activity performed throughout a day [25] (Figure 1).

### 2.3. Measures

The survey comprised 29 closed-ended and 3 open-ended questions separated into 5 sections (complete survey shown in the Supplementary File S1).

Section 1 and Section 2 included eligibility questions assessing participant characteristics and demographics using text entry, dichotomous (yes/no), and multiple-choice questions. Demographic questions (gender, income, education level, postcode) were adapted from the Australian Bureau of Statistics (Figure 1) [26].

### 2.4. Sports Food Consumption, Perceived Risks, and Side Effects Experienced

Section 3 questions included type of sports food consumed in the past three months (protein powder, carbohydrate gel, creatine, etc.), how often selected products were consumed (anchors: multiple times per day/everyday, once every three months or less), cost when last purchased (anchors: less than $20, $81–$100) and timing of consumption (anchors: before exercise, immediately after). For all sports foods selected in the multiple-response sports food type consumed question, participants were asked the open-ended question “Why do you consume ‘selected sports food’?”. If participants indicated they knew of perceived risks or had experienced side effects (e.g., are you aware of potential risks? have you experienced side effects?), they were asked to detail these via the open-ended questions “What sort of side effects have you experienced?” and “What potential risks are you aware of?” (Figure 1).

### 2.5. Exercise and Incidental Physical Activity Participation

In Section 4, questions asked about exercise and incidental physical activity participation were adapted from previous surveys [27]. Participants were asked whether they had participated in sports/exercise/incidental physical activity with the past 3 months, their activity type from a range of 14 pre-selected activities (e.g., field ball, weight training, active transport) and if this was the amount they normally did (e.g., more than you would usually do, about the same, less than you would usually do). For each selected activity, participants were asked about frequency (anchors: very infrequently; very frequently) and duration (anchors: 30 min or less; 3 h or more) for each identified activity undertaken (Figure 1).

### 2.6. Purchasing Patterns, Packaging Label Use, and Recommendations

Section 5 included questions related to purchase location (e.g., supermarket, chemist), who recommended the sports foods to participants (e.g., friends, family, media), the packaging attribute/s that were used when selecting the product, if any (e.g., written information, nutrition information), the amount of packaging used in the purchasing decision (e.g., all of the packaging information, most of the packaging information, some of the packaging information, none of the packaging information), and perceptions of the amount of information on the packaging (too much, about right, too little) (Figure 1).

### 2.7. Data Manipulation

The continuous variable for age was organised into age ranges (29 y and under, 30–49 y, 50 y and over). Two individual-level measures of socio-economic status were used (education, income); therefore, post code (an area level indicator) was not used for this analysis. The remaining 2 demographic variables were collapsed from ranges of 5–6 options into smaller ranges for more even distribution between groups for annual income (under 50,000, 50,000–149,000, 150,000 and above) and education (year 12 and above, trade certificate, bachelor’s degree or higher) levels. Participants could select multiple options from 14 predefined sports food product types, and these were grouped into ‘protein products’ selected (powder, bars, readymade shakes), ‘carbohydrate products’ selected (powders, gels), and all other products selected into the category of ‘other’ (creatine, glutamine, Branch Chain Amino Acid, beta-alanine, L-carnitine, pre-workout, fat burners, other). Participants could select multiple exercise types from predefined choices, and the number of exercise types selected by each participant were combined and grouped into two categories (exercise variety low up to 2 types, exercise variety moderate to high 3 or more types). The same method was used to determine incidental physical activity (low 1 type, high 2 or more types). For purchase location, health-food store/supplement store and gym/personal trainer were combined into separate variables. For source of recommendation, packaging claims or other statements and store-based employees were separate variables, and three variables were created from all remaining possible choices: friends/family/peer groups; personal trainer/gym reception; and digital/print/broadcast media.

Since actual sports food consumption was not measured, a consumption frequency proxy score was calculated. The number of sports foods consumed from each participant (range 1–14) was added together. The frequency of consumption of each selected product was allocated a count (very infrequently = 1 to very frequently = 5), and these counts were added to form a frequency count. The counts for the number of products consumed and frequency were then added to obtain a consumption/frequency proxy score (range 4–51). This proxy score was then separated into tertials (low ≤10, moderate 11–19, high 20+).

### 2.8. Data Analysis

All quantitative data were analysed using the Statistical Package for the Social Sciences (SPSS for Macintosh) version 29.0.0.0 (SPSS Inc., Chicago, IL, USA). Descriptive statistics (frequencies, percent) were used to describe the characteristics of the sample. Ordinal logistic regression was used to determine the odds ratio, confidence interval, and chi square significance for univariate associations. A multivariate model was used to determine relationships between sports food consumption frequency proxy categories (dependant variable) and significant univariate associations. Statistical significance was set at *p* < 0.05.

When designing this study, it was determined that the recruitment of 776 participants would provide 80% power to detect a minimum 10% difference in proportions between gender groups; however, the sample size of the final study was determined by the feasibility of recruitment, which was slow, and only 307 participants were recruited, allowing for 80% power to detect a minimally detectable effect size of 16%.

The open-ended questions were analysed using inductive thematic analysis in NVivo version 14 (QSR International Pty Ltd., Burlington, MA, USA, 2023). Coding was conducted by CC and AB for 10% of responses and was compared to determine if intercoder reliability was reached [28]. Discrepancies were discussed until intercoder agreement was reached and the remaining 90% of responses were coded by CC. Key themes were discussed again between CC and AB at the completion of coding to ensure agreement regarding all themes. The codes were grouped into overarching major and sub-themes.

## 3. Results

During the data-collection period, 531 participants accessed the survey, 83 were excluded for not meeting the inclusion criteria, and prior to data analysis the gender categories of non-binary (*n* = 7) and self-described (*n* = 1) were omitted due to the small sample size, resulting in 307 usable responses. The survey took an average of 22 min to complete.

### 3.1. Participant Characteristics

The sample was fairly evenly distributed across genders (females made up 56.4% of participants) and most (45.0%) were in the 30–49 years category with a range of 18–65 years. The sample was composed of participants from a range of socioeconomic positions. Just over half of consumers held a bachelor’s degree or higher (54.4%). Slightly more than half of consumers (53.4%) had an income in the range of AUD 50,000 to 149,000 per year (Table 1).

### 3.2. Sports Food Consumption, Perceived Risks, and Side Effects Experienced

Protein products were consumed by 94.5% of participants, followed by ‘other’ products (61.9%), and around one in five consumed carbohydrate products (19.2%). Participants consuming three or more products made up 64.5% of the sample, and the highest number of products consumed by a single participant was twelve (range 1–12). Just over half (52.4%) of the participants stated they were aware of risks of consuming sports foods and around a third (34.5%) stated they had experienced a side effect from using sports foods (Table 1).

### 3.3. Exercise, Incidental Physical Activity, and Walking Participation

Over half (58.3%) of the sample participated in up to two exercise types and over two thirds (67.0%) stated that they did weight training as one of the exercises performed. Walking was performed by 73.0% of participants. The majority of participants (64.8%) did one incidental physical activity.

### 3.4. Purchasing Patterns, Packaging Label Use, and Recommendations

The supermarket (51.5%), internet (48.9%), and health-food/supplement stores (38.1%) were the main locations where sports foods were purchased from by participants. The most common source by which sports foods were recommended to participants were digital/print/broadcast media (38.8%), with friends/family/peer groups (29.6%) and packaging claims (29.3%) also being commonly reported recommendations by others. The nutrition information panel (95.4%) and claims (40.1%) were stated as the most commonly used packaging attributes to select products (Table 1).

### 3.5. Factors Related to Sports Food Consumption

As shown in Table 2, females had significantly higher odds (OR 2.36 (1.43, 3.89) *p* < 0.001) of being in a higher sports food consumption/frequency category compared to males. Age, annual income, and education level were not associated with sports food consumption/frequency. In the univariate analysis, participants who did perceive risks (OR 0.40 (0.26, 0.61) *p* < 0.001) and/or experience side effects (OR 0.25 (0.16, 0.39) *p* < 0.001) had significantly lower odds of being in a higher consumption/frequency category compared to those who did not perceive risks or had not experienced side effects. However, this did not remain in the multivariate analysis for perceived risks. Those who participated in two or more incidental physical activity types had significantly lower odds (OR 0.52 (0.31, 0.86) *p* < 0.011) of being in a higher sports food consumption/frequency category in the multivariate model. Walking was significant in the univariate analysis (OR 0.57 (0.36, 0.91) *p* < 0.018) but did not remain so in the multivariate model, and the number of exercise types performed was not associated. Those who purchased from the supermarket (OR 0.32 (0.19, 0.52) *p* < 0.001), internet (0.26 (0.15, 0.45) *p* < 0.001), health-food/supplement store (OR 0.23 (0.14, 0.38) *p* < 0.001), or chemist (OR 0.40 (0.24, 0.66) *p* < 0.001) had significantly lower odds of being in a higher sports food consumption/frequency category in the multivariate model compared to those who did not purchase from these locations. Purchasing from a gym/personal trainer was not associated. In the univariate analysis, participants who were recommended sports foods by digital/print/broadcast media had lower odds of being in a high sports food consumption/frequency category (OR 0.53 (0.35, 0.81) *p* < 0.004); however, this did not remain in the multivariate analysis, and all other sources of recommendation were not associated with being in a higher sports food consumption/frequency category. The use of packaging attributes for selection was not associated with the consumption/frequency category (Table 2).

### 3.6. Open-Ended Question Themes

There were 17 major themes mentioned by participants for why they consumed sports foods, their perceptions of the risks of consumption, and any side effects that were experienced. The numbers and percentages of participants who reported each theme are in Table 3.

### 3.7. Why Sports Foods Are Consumed

There were seven major themes covering the reasons sports foods were consumed. The most mentioned were protein intake, muscle (sub theme: growth), exercise general (sub-theme: stamina/energy and recovery), and convenience (sub-theme: snack).

#### 3.7.1. Major Theme: Protein Intake

Many participants mentioned protein intake as a reason why they consumed sports foods, some simply stating ‘for protein’ (F, 35 years old (y)) or ‘for a high protein’ (M, 49 y). Other participants stated they consumed sports foods ‘to up my protein intake per day’ (F, 56 y) and ‘to reach daily protein targets’ (M, 51 y). Other participants made particular reference to inadequate meat intake in their diet and the need to supplement their protein intake, ‘I do not eat enough protein from meats’ (F, 35 y) and ‘I need more protein in my diet as I don’t eat much meat’ (F, 27 y).

#### 3.7.2. Major Theme: Muscle Any Mention

Muscle as a reason for sports food consumption ‘to aid in muscle function’ (M, 37 y) or ‘to assist muscles’ (F, 58 y) was mentioned frequently. Other participants stated the sub-theme ‘growth’ such as ‘for muscle growth’ (F, 37 y) and ‘to give my muscles the after work out nutrients and proteins to get bigger, stronger and better’ (M, 35 y). Additionally, the sub-theme ‘repair’ was also present, with participants stating: ‘to help muscle repair’ (M, 24 y) and ‘facilitate muscle repair’ (F, 23 y).

#### 3.7.3. Major Theme: Exercise General

There were many mentions that consuming sports foods was beneficial for exercise, generally and more specifically for performance, recovery, stamina, energy, and improved training, by participants. For instance, ‘assist with intense exercise’ (F, 18 y) and ‘lift heavier in the gym’ (M, 39 y) as ‘general exercise’ reasons for why sports foods were consumed. For the sub-theme ‘performance’, consumers mentioned, ‘to aid in my performance’ (M, 37 y) or ‘to enhance performance’ (F, 32 y). Specific mentions of ‘to help with muscle recovery’ (F, 28 y) or simply ‘recovery’ (F, 51 y) for the sub-theme ‘recovery’ were stated by participants. Other mentions for the sub themes ‘stamina, energy’ as ‘to help with stamina and ability’ (F, 28 y) and ‘for an extra boost of energy on days I’m feeling exhausted but need to maintain high energy levels for work’ (M, 30 y) were made. Some participants referred to the sub-theme ‘training, workout’ under the major theme ‘exercise general’ as ‘to maximise training capacity’ (M, 37 y) and ‘to encourage me to go workout on days I don’t feel like it’ (M, 62 y).

#### 3.7.4. Major Themes: Convenience

Convenience was also an important theme driving sports food consumption. Participants mentioned that ‘they are convenient’ (F, 29 y) or ‘for convenience when busy or working or on the go’ (M, 54 y), as a reason for consuming sports foods. Other participants made particular reference to the sub theme ‘snack’; ‘snack during the day’ (M, 31 y) and ‘filling snack compared to similar items’ (F, 58 y).

#### 3.7.5. Less Frequent Major Themes: Weight Loss, Tastes Good, and Healthy Choice

Another perceived benefit of consuming sports foods was for weight loss. Participants stated, ‘to assist with weight loss’ (M, 48y), or simply ‘get lean’ (F, 18 y), and statements such as ‘to promote fat loss and help me lose weight’ (F, 35 y), as reasons why they consumed sports food products. Participants also referred to ‘healthy choice when I have nothing else prepared’ (M, 52 y), ‘they are much healthier than chocolate bars’ (M, 43 y), and simply ‘good for my health’ (F, 48 y) as reasons why sports foods were consumed. The major theme of taste was mentioned by participants, with statements such as ‘taste nice’ (F, 60 y) and ‘to make water taste better’ (M, 34 y).

### 3.8. Perceived Risks

There were three major themes mentioned by participants as perceived risks of sports food consumption. The most frequently mentioned health issues (sub theme: digestion and stomach issues, kidney/liver/organ issues, heart, and cardiovascular issues.

#### 3.8.1. Major Theme: Health Issues

For the sub-theme ‘digestion and stomach’, participants mentioned simply ‘causes bloating’ (F, 28 y), ‘nausea’ (F, 34 y), and ‘laxative/constipating effect’ (M, 49 y) as some of the perceived risks of consuming sports foods. For the sub theme ‘kidney, liver, organ issues’, participants stated ‘too much protein can be bad for kidneys’ (M, 64 y), ‘potential liver damage’ (F, 29 y), and ‘organ issues’ (F, 45 y). Participants mentioned ‘heart palpitations’ (M, 62 y), ‘high blood pressure’ (M, 37 y), and ‘potential for a heart attack’ (F, 48 y) under the sub theme ‘heart and cardiovascular issues’.

#### 3.8.2. Less Frequent Major Themes: Caffeine Issues and Contamination

Some participants mentioned ‘too much caffeine’ (F, 22 y), ‘caffeine overdose’ (M, 39 y), and ‘too much caffeine can be problematic’ (M, 49 y) as perceived risks of consuming sports foods. Other perceived risks mentioned by participants were ‘contamination’ (F, 23 y), ‘contamination with banned substances’ (F, 29 y), and ‘poor regulation of products leading to contamination’ (M, 63 y).

### 3.9. Side Effects Experienced

There were seven major themes mentioned by participants in relation to the side effects they had experienced. The most frequently mentioned major and sub-themes were digestion and stomach issues, heart/cardiovascular issues, and jitters/shaking/trembling.

#### 3.9.1. Major Theme: Health Issues

Participants specifically mentioned the sub-theme ‘digestion and stomach issues’ under health issues and stated ‘diarrhoea’ (F, 32 y) and ‘when using regularly I would get mild stomach pains’ (M, 30 y). Other participants mentioned health issues such as the sub-theme ‘heart and cardiovascular issues’ and stated, ‘increased heart rate’ (F, 42 y) and ‘the wrong one, chest pains’ (M, 44 y) as side effects experienced. Additionally, the health-issue sub-theme of ‘intolerances’ was mentioned by participants as ‘lactose intolerance’ (F, 30 y) and ‘adverse effects for fructose intolerance’ (M, 18 y).

#### 3.9.2. Major Theme: Anxiety

Participants mentioned the major theme of ‘anxiety’ as a side effect experienced and stated simply ‘anxiety’ (F, 29 y) and ‘anxiety attacks’ (F, 29 y). Other participants mentioned the sub-theme ‘jitters, shaking, tingling’ as anxiety-related issues and said ‘jitters’ (F, 58 y), ‘some shaking’ (M, 49 y), and ‘tingling in hands and face’ (F, 42 y).

#### 3.9.3. Less Frequent Major Themes: Lightheaded/Dizzy/Headaches, Sleep Disturbance, Overheating/Sweating/Dehydration, Caffeine Issues, and Skin Issues

Side effects experienced under the major theme lightheaded, dizzy, and headaches were mentioned by participants as ‘light headedness’ (M, 62 y), ‘dizziness, head spin’ (M, 31 y), and ‘really bad headaches’ (F, 35 y). Sleep disturbance was mentioned as a side effect of sports food consumption as ‘sleepless nights’ (M, 44 y) and ‘insomnia’ (F, 42 y). For the major theme ‘overheating, sweating and dehydration’, participants mentioned ‘overheating’ (M, 64 y), ‘overly sweaty’ (F, 56 y), and ‘dehydration’ (M, 18 y), as side effects from sports food consumption. Side effects related to the sub-theme of caffeine issues were mentioned by participants as ‘over caffeinated’ (F, 42 y) and ‘feeling sometimes if I’ve also had a lot of caffeine’ (F, 39 y). One participant made particular reference to a change in the body as ‘jaw clenching which could be caused by the caffeine content’ (M, 65 y). When mentioning the side effect of skin issues, participants stated ‘itchy skin’ (F, 48 y) and ‘rash’ (F, 32 y).

## 4. Discussion

The aims of the present study were to describe the consumption patterns, social ecological factors, and drivers associated with sports food consumption in non-athletes, and to understand the reasons for their consumption and the perception of risks and side effects by the non-athlete Australian sports food consumer. In relation to consumption patterns, the key findings were that females were more likely than males to consume more sports foods and multiple participants were stacking products. Regarding the reasons for consumption, protein intake was reported as the major reason for consuming sports foods. Participants who consumed more sports foods were less likely than those with lower consumption to perceive risks associated with consuming sports foods or to experience side effects. The major perceived risks of consuming sports foods, when reported, were digestion/stomach issues and kidney/liver/organ issues. The major side effects reported were stomach/digestion issues and jitters/shaking/trembling.

Sports food consumption patterns differed according to the gender of the participants, and there has been an observed shift in the main consumers of these foods since data were last collected over 10 years ago. The present study found that females were the predominant consumers of sports foods. However, two Australian studies have found contrasting relationships with gender [16,29]; the first, conducted in 2013, found that males (52%) were the predominant consumers of sports foods, while more recently, a 2023 study, examining the use of and attitudes towards sports foods, found consumers were more likely to be female (63.6%). However, the 2013 study was completed at a time when the sports food market was less developed than the present study [16], and the 2023 study included participants classified as ‘athletes’ or ‘exercisers’, which was not well defined. The study focused on the perceptions of the level of processing of sports foods rather than the foods themselves and perceptions and reasons for consumption [29], which differed to this present study. It is unclear exactly why there has been a shift in sports food consumers; however, it could be related to the intersection between gender differences in health beliefs, where females rate ‘health’ as a more important factor when making sports food choices [30]. Furthermore, it could be driven by an increased perception that supplementation is needed to ensure adequate nutrient levels, likely driven by the aggressive marketing of sports foods to non-athletes via social media channels [31] promoting the use of these products to this population.

The stacking of multiple sports foods was a key finding in the present study and is an issue which could lead to a range of health consequences. When multiple sports food products are consumed simultaneously, non-athletes could consume excess nutrients and experience unwanted side effects [10]. A finding of this study was that over two thirds (65.5%) of participants reported stacking sports food products which is in line with previous studies which suggested that the stacking of products was occurring within the population of general sports food consumers [16] and in athletes at an international level [11]. While stacking may, in the case of athletes, provide some benefits to performance [32,33], these are relevant only to elite and high-performance athletes because of the greater need for energy due to high exercise output and not to the general non-athlete population. There is cause for concern as non-athlete consumers could be inadvertently doubling up on nutrients, which could contribute to exceeding the recommended daily intake or the Upper Level of intake [9], effecting overall health status [34]. Additionally, consumers could be ingesting nutrients from different sources that should not be combined (e.g., high-iron food, iron supplements, or pre workout foods), increasing the risk of poor nutrient absorption by combining nutrients with other components (e.g., iron and caffeine) [10]. There is also the possibility that non-athletes are consuming substances prohibited by sport [35,36], such as banned stimulants that could be harmful and lead to serious health consequences. The Australian supplement survey conducted in 2016 found that 19% of the supplements purchased from both the internet and physical stores in Australia contained one or more substances which would be considered as prohibited by sport [37]. These substances included stimulants and anabolic agents, for which the health implications are unclear in this population and could be problematic for non-athlete consumers.

Participants mentioned the desire to increase protein intake as a predominant motivation for consuming sports foods. It is unclear why participants wanted to increase protein intakes, as national survey results indicate that Australians not only meet but exceed recommendations [38]. Similarly, studies examining dietary-supplement use found that consumers had adequate nutrient intakes through dietary sources yet were consuming supplements and exceeding the upper limits for some nutrients [39]. An Australian study also found that consumers of dietary supplements were generally in good health and consuming adequate nutrients through their diets [40]. These studies are limited though, by the examination of dietary supplements and not sports foods, for which there is a greater level of regulation in Australia and tighter restrictions on the packaging labels allowed to be displayed [39,40]. Potential drivers for protein intake being a predominant reason for sports food consumption could be the view that protein is deficient in the diet, perpetrated by an increased use of social media by general populations to obtain fitness and nutrition information [31]. Sports food marketing through digital social networks has increased, with research suggesting that fitness and coaching influencer pages have the highest number of followers and engagement by followers [31]. Protein-dominant sports foods in particular are being heavily marketed through social media pages related to fitness and nutrition content [31]. It is possible that this type of marketing is encouraging the use of sports foods by non-athletes and could subsequently be contributing to the increased use of packaging labels in the decision-making process. The increased use of packaging labels such as nutrition information panels or claims could be used as a tool to determine the level of nutrients in the food, driven by what has been marketed to them.

Nutrition information and claims can be influential sources for making purchasing decisions about sports foods, the present study finding that nutrition information panels and claims were used the most frequently to select sports food products. Previous research on the use of nutrition information panels for general foods found that a third of consumers use these when selecting food for the first time [41] and subsequent selection occasions [42]. Yet, the key reason that consumers claim to use nutrition labels is for finding specific nutrients in food products [43]. However, studies that examine the influence of claims of food selection tend to have conflicting findings [44,45,46]. Some research suggests that displaying claims increases the perception of healthiness of food products [44,45], while other studies found that claims were found to be untrustworthy, and other front of package-labelling schemes were more likely to be used [46]. A limitation of these studies is that they mostly examine individual packaging labels in isolation [41,42,43,44,45], which does not translate into a real-world setting where multiple labelling elements would be present. Furthermore, unlike the regular foods examined in the studies, sports foods are unable to display interpretive front-of-pack labelling schemes such as the Health Star Rating [47], and therefore it is unclear what the effect of claims alone are on selection of sports food products [46]. The use of nutrition information and claims to make purchasing decisions around sports foods is problematic as research suggests that not only can sports food nutrition information be inaccurate [12], but the inaccuracy may lead to claims being misleading [24,48]. In the case of sports foods, research suggests that misleading on-pack advertising practices could be a contributing factor in their misuse [24]. However, that study only examined protein products with a focus on the composition of these, which does not account for the range of other sports food products and label inaccuracies on products available in the current market. Current regulations for the labelling of sports foods in Australia donot restrict the number or types of claims and other information on labels, and it appears that the enforcement of regulations is lacking, given the inaccuracies found.

Understanding the perception of risk involved with consuming sports food products and the side effects experienced is an important factor to consider in the choice of these foods. This can be vital in determining the reasons why sports foods are being consumed by non-athletes. The present study found that those who consumed more sports foods were less likely to perceive any consumption risks or experience side effects, and participants mentioned a range of perceived risks and side effects such as digestion and kidney or liver issues and side effects such as jitters and shaking. The previous literature examining harm beliefs related to risk perception suggests that people tend to compare their risk as very low or lower to other similar people [49], and while initial decision-making anxiety is reduced in the short term, due to the underestimation of risks, it can be harmful in the long term [50]. Research examining perceived risks using packaging warnings on discretionary foods choice and the perception of diet-related health issues risks [51,52] found that consumers can be made aware of the risks of consumption using warnings; however, awareness is still dependant on the perception of the risk [51]. Additionally, in terms of dietary health issues, events that are perceived by consumers as controllable are generally perceived as being more favourable for taking the risk [52]. In previous research, when asked about the perceived risks of using sports foods, consumers determined them to be safe due the belief that the benefits outweigh the risks [53], and that health and wellbeing beliefs are more important in selection than the potential risks [54]. A key driver of lower risk perception and experiences of side effects could be the belief that sports food products are safe to consume due to their wide availability in mainstream retail settings, or that these foods are highly regulated, considering they are specialised foods. Furthermore, the lack of side effects experienced by participants consuming more sports foods could be related to the perception that these are side effects related to other foods being consumed and that the benefits of consumption are greater than the side effects experienced. However, it is difficult to determine where the lack of precaution and continued consumption of sports foods after risks and side effects have been identified stems from.

### 4.1. Implications and Future Research

Ensuring that there is detailed and clear information for consumers on the safety of products and advice provided around how these products should be consumed is imperative. Therefore, developing an understanding of the drivers and reasons for more frequent consumption is important to enable a clearer picture of why more females are consuming sports foods more frequently, to enable fit-for-purpose recommendations on sports food intakes and reduce the stacking of sports foods in this population in Australia. Future research should aim to understand why these risks are disregarded by consumers to provide knowledge on how to better label products to ensure a greater level of risk information is provided to consumers and lower the risk of experiencing unwanted side effects.

### 4.2. Strengths and Limitations

The strengths of this study are that it provides contemporary information regarding the demographic characteristics of sports food consumers, patterns of consumption, and the exercise participation of non-athlete sports food consumers in Australia. Furthermore, the sample in the study had a good spread of socioeconomic groups and was not dominated by one particular group. The study also provides an understanding of why consumers are using these products and how they perceive the risks and if they have experienced side effects, which has previously not been examined in detail. Furthermore, the inclusion of the open-ended questions enabled a clearer understanding of the factors influencing sports food consumption in this population.

However, the study is limited by the survey design, in which true consumption levels and exercise participation were not able to be determined, and this made the analysis of relationships more difficult. In addition, the sample size was relatively low compared to other surveys, and it was difficult to reach the desired sample size. The study therefore had diminished power to detect differences between factors.

## 5. Conclusions

There has been a shift in the type of consumer of sports food products over the past decade away from predominantly males to females. Non-athletes primarily consume sports foods for additional protein intake. Additionally, a number of health issues related to the perceived risks and side effects were mentioned. Sports food consumers are also using packaging information to determine the nutrients in foods, despite this information being found to at times be inaccurate. Yet, many non-athletes are consuming multiple products, mostly protein products, and could be consuming excess nutrients or contaminated products. Stronger packaging regulations including restrictions on the ways that sports foods can be marketed through media, especially social media channels, is needed. This will ensure that adequate information is provided to make informed and safe food choices. Future research that provides a deeper level of insight into the reasons why specific sports foods are consumed and what the perceived benefits of sports food consumption are, will aid in the development of public health communication strategies and regulations to ensure sports foods are consumed safely by non-athletes. Additionally, understanding more about how consumers perceive regulations, warnings, and risks and side effects could enable clear next steps in protective labelling practices and advice seeking for the safe consumption of sports food products by non-athletes.

## Figures and Tables

**Table 1:** Characteristics of sample of non-athlete Australian adult sports food consumers (*n* = 307).

Characteristic	*n* (%)
Gender	
Male	134 (43.6)
Female	173 (56.4)
Age (years)	
29 and under	88 (28.7)
30–49	138 (45.0)
50 and over	81 (26.4)
Annual income (AUD)	
Under 50,000	126 (41.0)
50,000–149,000	164 (53.4)
150,000 and above	17 (5.5)
Education level	
Year 12 and under	69 (22.5)
Trade certificate	71 (23.1)
Bachelor’s degree or higher	167 (54.4)
Sports food types consumed ^1^	
Protein	290 (94.5)
Other	190 (61.9)
Carbohydrate	59 (19.2)
Number of sports foods products used by consumers	
Up to two products	109 (35.5)
Three or more products	198 (64.5)
Perceived any risks of consuming all sports food types	
No	146 (47.6)
Yes	161 (52.4)
Any side effects experienced by consuming all sports food types	
No	201 (65.5)
Yes	106 (34.5)
Number of exercise types, incidental physical activity and walking performed	
Exercise variety: low (up to two types)	179 (58.3)
Exercise variety: moderate to high (three or more types)	128 (41.7)
Incidental physical activity: low (one incidental physical activity)	199 (64.8)
Incidental physical activity: high (two or more types)	108 (35.2)
Walking	224 (73.0)
Purchase location ^1^	
Supermarket	158 (51.5)
Internet	150 (48.9)
Health-food/supplement store	143 (46.6)
Chemist	113 (36.8)
Gym/personal trainer	19 (6.2)
Source by which all sports food types were recommended to participants ^1^	
Digital, print, and broadcast media	119 (38.8)
Friends/family/peer groups	91 (29.6)
Packaging claims or other statements	90 (29.3)
Personal trainer/gym reception	58 (18.9)
Store-based employee	39 (12.7)
Product label elements used when making purchasing decision ^1^	
Nutrition information panel	293 (95.4)
Claims	123 (40.1)
Warning and/or advisory statements	95 (30.9)
Imagery and colour	40 (13.0)

^1^ Multiple responses permitted.

**Table 2:** Sports food consumption (proxy) and association with demographic, perceived risks, side effects experienced, exercise type, purchase location, recommendation source, and product label used for selection in Australian adult non-athletes (*n* = 307).

	Sports Food Consumers			
	Univariate Model		Multivariate Model	
Characteristics	Odds Ratio (95% CI)	*p* Value	Odds Ratio (95% CI)	*p* Value
Gender				
Male (reference)	1.00		1.00	
Female	1.82 (1.19, 2.76)	0.005	2.36 (1.43, 3.89)	<0.001
Age in years (range)				
29 and under (reference)	1.00			
30–49	1.18 (0.73, 1.92)	0.49		
50 and over	1.57 (0.90, 2.76)	0.12		
Annual income				
Under 50,000 (reference)	1.00			
50,000–149,000	0.87 (0.57, 1.34)	0.53		
150,000 and above	0.67 (0.28, 1.62)	0.38		
Education level				
Year 12 and under (reference)	1.00			
Trade certificate	0.72 (0.32, 1.33)	0.30		
Tertiary degree	1.08 (0.65, 1.80)	0.76		
Perceived risks of consuming all sports food types				
No (reference)	1.00		1.00	
Yes	0.40 (0.26, 0.61)	<0.001	0.64 (0.40, 1.04)	0.07
Side effects experienced				
No (reference)	1.00		1.00	
Yes	0.25 (0.16, 0.39)	<0.001	0.28 (0.17, 0.48)	<0.001
Number of exercise types performed				
Up to two types (reference)	1.00			
Three or more types	0.66 (0.44, 1.00)	0.05		
Number of incidental exercises performed				
One incidental exercise type (reference)	1.00		1.00	
Two or more incidental exercise types	0.51 (0.33, 0.78)	0.002	0.52 (0.31, 0.86)	0.011
Walking performed				
No	1.00		1.00	
Yes	0.57 (0.36, 0.91)	0.02	0.85 (0.49, 1.48)	0.56
Purchased from				
Supermarket				
Did not (reference)	1.00		1.00	
Did	0.54 (0.36, 0.82)	0.004	0.32 (0.19, 0.52)	<0.001
Internet				
Did not (reference)	1.00		1.00	
Did	0.43 (0.29, 0.65)	<0.001	0.26 (0.15, 0.45)	<0.001
Health food/supplement store				
Did not (reference)	1.00		1.00	
Did	0.30 (0.20, 0.47)	<0.001	0.23 (0.14, 0.38)	<0.001
Chemist				
Did not (reference)	1.00		1.00	
Did	0.58 (0.38, 0.89)	0.01	0.40 (0.24, 0.66)	<0.001
Gym/personal trainer				
Did not (reference)	1.00			
Did	0.54 (0.24, 1.20)	0.13		
Recommended by				
Digital/print/broadcast media				
Not recommended (reference)	1.00		1.00	
Recommended	0.53 (0.35, 0.81)	0.004	0.75 (0.46, 1.21)	0.24
Friends/family/peer groups				
Not recommended (reference)	1.00			
Recommended	0.74 (0.47, 1.16)	0.19		
Packaging claims or other statements				
Not recommended (reference)	1.00			
Recommended	1.02 (0.64, 1.61)	0.95		
Personal trainer/gym reception				
Not recommended (reference)	1.00			
Recommended	0.67 (0.40, 1.12)	0.13		
Store-based employee				
Not recommended (reference)	1.00			
Recommended	0.65 (0.34, 1.24)	0.19		
Product label used for selection				
Nutrition information panel				
Did not use	1.00			
Did use	0.47 (0.17, 1.27)	0.14		
Claims				
Did not use	1.00			
Did use	1.08 (0.71, 1.64)	0.73		
Warning/advisory statement				
Did not use	1.00			
Did use	0.85 (0.54, 1.33)	0.48		
Imagery and colour				
Did not use	1.00			
Did use	1.15 (0.62, 2.12)	0.65		

**Table 3:** Themes, sub-themes, total number, and percent of mentions for why sports foods are consumed, perceived risks, and side effects experienced for sports food consumption.

Major Theme	Sub-Theme	Mentions
Why sports food products consumed (*n* = 307)		
Protein intake	-	174 (56.7)
Muscle	-	11 (3.6)
-	Growth	109 (35.5)
-	Repair	37 (12.1)
Exercise general	-	10 (3.3)
-	Stamina/energy	107 (34.9)
-	Recovery	104 (33.9)
-	Training/workout	40 (13.0)
-	Performance	37 (12.1)
Convenience	-	56 (18.2)
-	Snack	61 (19.9)
Healthy choice	-	39 (12.7)
Weight loss	-	47 (15.3)
Tastes good	-	43 (14.0)
Perceived risks (*n* = 142)		
Health issues	-	-
-	Digestion/stomach issues	70 (49.3)
-	Kidney/liver/organ issues	54 (38.0)
-	Heart/cardiovascular issues	38 (26.8)
Caffeine issues	-	22 (15.5)
Contamination	-	19 (13.4)
Side effects experienced (*n* = 118)		
Health issues	-	-
-	Digestion/stomach issues	91 (77.1)
-	Heart/cardiovascular issues	12 (10.2)
-	Intolerances	3 (2.5)
Anxiety	-	7 (5.9)
-	Jitters/shaking/tingling	21 (17.8)
Lightheaded/dizzy/headaches	-	9 (7.6)
Sleep disturbance	-	9 (7.6)
Overheating/sweating/dehydration	-	7 (5.9)
Caffeine issues	-	5 (4.2)
Skin issues	-	5 (4.2)

## Data Availability

The raw data supporting the conclusions of this article will be made available by the authors on request.

## Supplementary Materials

The following supporting information can be downloaded at https://www.mdpi.com/article/10.3390/nu16081101/s1, File S1: Formulated supplementary sports food choice and consumption population survey.
