# Supplementary material for: A Cross-Sectional Study of Sports Food Consumption Patterns, Experiences, and Perceptions amongst Non-Athletes in Australia
Source: Nutrients. 2024 Apr 9;16(8):1101. doi: 10.3390/nu16081101 (PMC11053821; doi:10.3390/nu16081101)
Supplement: Supplementary file 1 [file nutrients-16-01101-s001.zip › nutrients-2931924-supplementary.pdf]

Online survey: Formulated supplementary sports food choice and consumption population survey

Section 1: Eligibility criteria

Thank you for your interest in participating in this survey.

The survey is being conducted to understand consumption and purchase of **formulated sports foods** in the Australian non-athlete population and is part of a PhD project being undertaken by Celeste Chapple at Deakin University.

The survey should take around 15 minutes to complete.

Please read the Plain Language Statement below.

Once you understand what the project is about, click next, this will be taken as your agreement to participate.

**PLS included in Appendix 3*

Please click **next** to answer a few questions to determine if you are eligible to participate in the survey. If you are eligible, you will continue to the main study.

Page break --

1 What is your age in years:

Answer ________________

**If age in years is under 18 years or over 65 years*

Unfortunately, you are not eligible to complete this survey.
Thank you for your time.

**If age in years is between 18-65 years old, continue to question 1.2*

Page break --

*The following questions will be about **formulated sports foods.** These are products specially formulated to help people achieve specific nutritional or performance goals, such as providing energy, assisting endurance or recovery after sport, or building muscles and strength.*

Formulated sports foods include:

- *Protein powders, protein bars, protein snacks (cookies, chocolates) and Ready To Drink (RTD) protein shakes.*

- *Carbohydrate powders and gels.*
- *Energy supplements such as branch chain amino acid (BCAA) powders, pre workout, beta alanine, L-carnitine, creatine, glutamine and/or fat burners.*

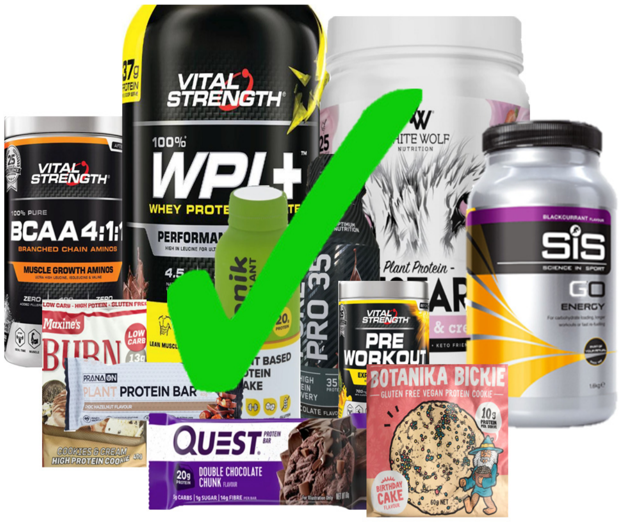

Formulated sports foods ***DO NOT*** *include:*

- *Ready-made sports or energy drinks such as vitamin waters, Gatorade, Powerade or Red Bull*
- *General food items such as meals made from meat, vegetables, and dairy products*
- *Tablets, capsules, or caplets*
- *Fruit*
- *Smoothies made from fruit and vegetables*

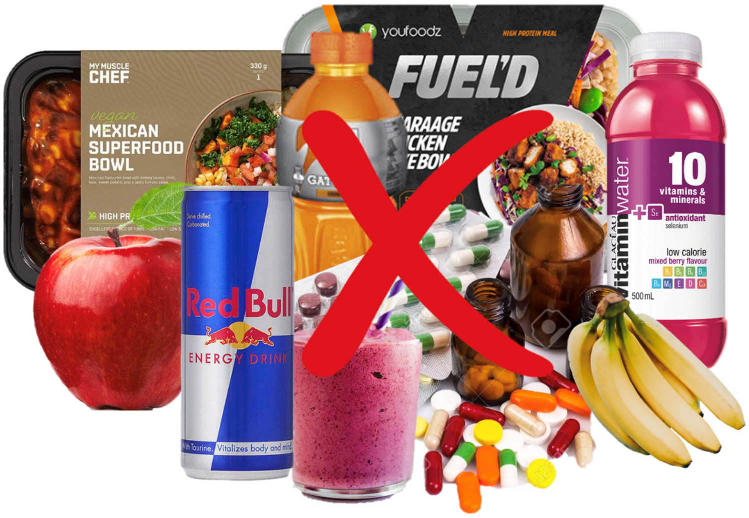

1.1 Have you consumed any formulated sports foods within the past 3 months?

- o Yes
- o No

**If answered 'no'*

Unfortunately, you are not eligible to complete this survey.

Thank you for your time

**If answered 'yes' continue to question 1.2*

Page break --

1.2 Do you work as an employee of a company making/selling formulated sports foods?

- o Yes
- o No

1.3 Are you sponsored by a company that makes/sells formulated sports foods (e.g. social media)?

- o Yes
- o No

1.4 Do you consume formulated sports foods under the ongoing monitoring of a dietitian or nutritionist?

- o Yes
- o No

**If answered 'yes' to questions 1.2, 1.3 and/or 1.4*

Unfortunately, you are not eligible to complete this survey.

Thank you for your time

**If answered 'no' to questions 1.2, 1.3 and/or 1.4, continue to question 1.5*

Page break --

1.5 Are you currently participating in a sport/sports/exercise/physical activity aiming to improve your performance or results?

- Yes
- No

1.6 Are you actively participating in sport/sports/exercise/physical activity competitions?

- Yes
- No

1.7 Are you formally registered in a local, regional, national, or international sport federation as a competitor?

- Yes
- No

1.8 Is sport participation and competition your major daily activity or focus of interest, almost always devoting several hours in all or most of the days in the week to these sport activities?

- Yes
- No

**If answered 'no' to questions 1.5, 1.6. 1.7 and/or 1.8*

You are eligible to take part in this survey.

**If answered 'yes' to questions 1.5, 1.6. 1.7 and 1.8*

Unfortunately, you are not eligible to complete this survey.

Thank you for your time

Page break --

Section 2: Demographics

2.1 What is your gender?

- Male
- Female
- Non-binary
- Self-described ________________

Page break --

2.2 What is your home postcode?

Answer ________________

Page break --

2.3 What is the highest educational qualification you have completed?

- No formal qualification
- Year 10 equivalent (e.g. school certificate)
- Year 12 equivalent (e.g. Higher school certificate)
- Trade/Certificate (e.g. hairdresser, chef, tradesperson)
- Undergraduate university degree (e.g. Bachelor)
- Postgraduate university degree (e.g., Masters/PhD)

Page break --

2.4 Before tax, which of the following best describes your annual personal income range from all sources?

- Less than $10,000
- $10,000 - $29,999
- $30,000 - $49,999
- $50,000 - $79,999
- $80,000 - $149,999
- More than $150,000

Page break --

Section 3: Sports food consumption:

*The following questions relate to **formulated sports foods** that you have consumed within the last three months.*

These questions will ask you about the type of sports foods you have consumed, the frequency at which you consume them , whether you consume the products according to the recommended servings displayed on the packaging, when you consumed these products and whether you experienced any problems, side effects or were aware of any potential risks from consuming the products.

There are three main categories of formulated sports foods:

1. *Protein*
2. *Carbohydrate*
3. *Energy*

Page break --

The images below are **examples** of formulated sports foods to understand the type of products within each category. These are **NOT meant to be** the exact products you have consumed.

3.1 Which types of formulated sports foods have you consumed within the past 3 months?

(Multiple choices can be selected)

- o **Protein powder**

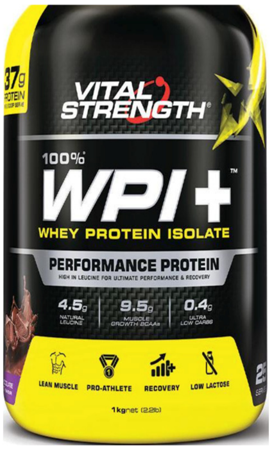

- o **Protein Bar**

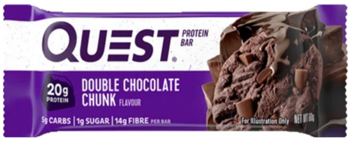

- **Protein snacks (chocolate, cookies etc)**

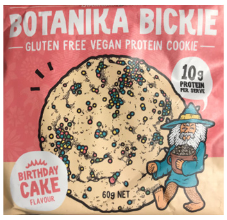

- **Protein Ready-To-Drink (pre-made drinks)**

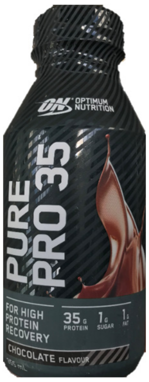

- **Carbohydrate gels**

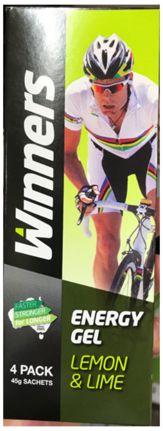

- **Carbohydrate powder (e.g. maltodextrin powder)**

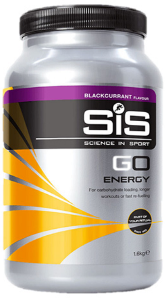

- Energy: Branch Chain Amino Acid powder (BCAA)

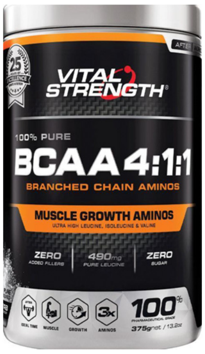

- Energy: Creatine powder

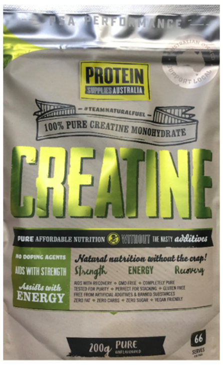

- Energy: Glutamine powder

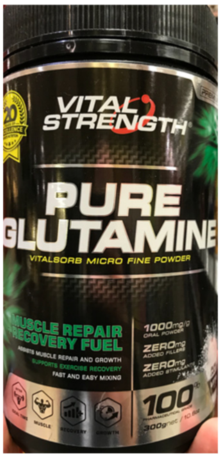

- Energy: Beta alanine powder

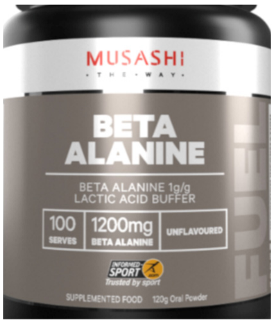

- Energy: L-Carnitine powder

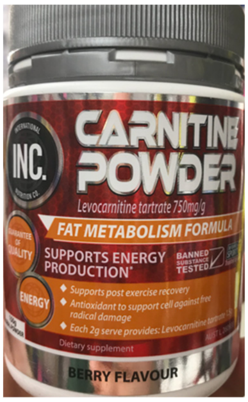

- Energy: Pre-workout powder

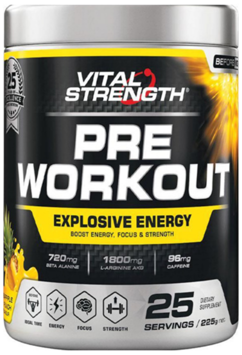

- Energy: Fat burners

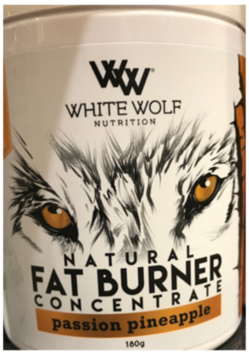

- Other (please specify)

Page break --

3.2 How often consumed In the last 3 months, how often have you consumed each of the following **formulated sports foods**? *(Please select one answer per product)*

	Very frequently (multiple times per day, every day)	Frequently (Once per week or two weeks)	Infrequently (Once a month)	Very infrequently (Once every three months or less)	Never
**Protein powder**	○	○	○	○	○
**Protein bar**	○	○	○	○	○
**Protein snacks (cookies, chocolate etc.)**	○	○	○	○	○
**Protein Ready-To-Drink (pre-made drinks)**	○	○	○	○	○
**Carbohydrate gel**	○	○	○	○	○
**Carbohydrate powder (e.g. maltodextrin)**	○	○	○	○	○
**Branch Chain Amino Acid powder (BCAA)**	○	○	○	○	○
**Creatine powder**	○	○	○	○	○
**Glutamine powder**	○	○	○	○	○
**Beta alanine**	○	○	○	○	○
**L-Carnitine powder**	○	○	○	○	○
**Pre-workout**	○	○	○	○	○
**Fat burners**	○	○	○	○	○

Other (please specify)	○	○	○	○	○

Page break ---

3.3 When consumed Which of the following best describes when you would usually consume **formulated sports foods**? *(Please tick all that apply)*

	Immediately Before physical activity	During physical activity	Immediately After finishing physical activity	Other times
**Protein powder**	☐	☐	☐	☐
**Protein bar**	☐	☐	☐	☐
**Protein snacks (cookies, chocolate etc)**	☐	☐	☐	☐
**Protein Ready-To-Drink (pre made drinks)**	☐	☐	☐	☐
**Carbohydrate gel**	☐	☐	☐	☐
**Carbohydrate powder (e.g. maltodextrin)**	☐	☐	☐	☐
**Branch Chain Amino Acid powder (BCAA)**	☐	☐	☐	☐
**Creatine powder**	☐	☐	☐	☐
**Glutamine powder**	☐	☐	☐	☐
**Beta alanine**	☐	☐	☐	☐
**L-Carnitine powder**	☐	☐	☐	☐
**Pre-workout**	☐	☐	☐	☐
**Fat burners**	☐	☐	☐	☐

Other (please specify)	☐	☐	☐	☐

** If 'other times' selected in any column in question 3.4, display question 3.5*

Page break --

3.4 Which other times have you used formulated sports foods for?

Answer _________________

Answer _________________

Answer _________________

Answer _________________

Page break --

3.5 Cost When you last purchased the previously selected **formulated sports foods**, how much did you spend on each product within each category?

(Round to the nearest dollar)

	Less than $20	$21-$40	$41-$60	$61-$80	$81-$100	More than $100
**Protein powder**	○	○	○	○	○	○
**Protein bar**	○	○	○	○	○	○
**Protein snacks (cookies, chocolate etc.)**	○	○	○	○	○	○
**Protein Ready-To-Drink (pre-made drinks)**	○	○	○	○	○	○
**Carbohydrate gel**	○	○	○	○	○	○
**Carbohydrate powder (e.g. maltodextrin)**	○	○	○	○	○	○
**Branch Chain Amino Acid powder (BCAA)**	○	○	○	○	○	○
**Creatine powder**	○	○	○	○	○	○
**Glutamine powder**	○	○	○	○	○	○
**Beta alanine**	○	○	○	○	○	○
**L-Carnitine powder**	○	○	○	○	○	○
**Pre-workout**	○	○	○	○	○	○
**Fat burners**	○	○	○	○	○	○
Other (please specify)	○	○	○	○	○	○

Page break --

**If protein powder in question 3.1 is selected, display question 3.6*

3.6 Why do you consume protein powder?

Answer ________________

3.7 Have you personally experienced any problems or side effects from consuming protein powder?

- o Yes
- o No

**If 'yes' selected display question 3.8*

**If 'no' selected display question 3.9*

3.8 What sort of side effects have you experienced?

Answer ________________

3.9 Are you aware of any potential risks associated with consuming protein powder?

- o Yes
- o No

**If 'yes' selected display question 3.10*

**If 'no' selected display question 3.11*

3.10 What potential risks are you aware of?

Answer ________________

**If protein bars in question 3.1 is selected display question 3.11*

3.11 Why do you consume protein bars?

Answer ________________

3.12 Have you personally experienced any problems or side effects from consuming protein bars?

- o Yes
- o No

**If 'yes' selected display question 3.13*

**If 'no' selected display question 3.14*

3.13 What sort of side effects have you experienced?

Answer ________________

3.14 Are you aware of any potential risks associated with consuming protein bars?

- o Yes
- o No

**If 'yes' selected display question 3.15*

**If 'no' selected display question 3.16*

3.15 What potential risks are you aware of?

Answer ________________

**If protein snacks in question 3.1 is selected display question 3.16*

3.16 Why do you consume protein snacks?

Answer ________________

3.17 Have you personally experienced any problems or side effects from consuming protein snacks?

- o Yes
- o No

**If 'yes' selected display question 3.18*

**If 'no' selected display question 3.19*

3.18 What sort of side effects have you experienced?

Answer ________________

3.19 Are you aware of any potential risks associated with consuming protein snacks?

- o Yes
- o No

**If 'yes' selected display question 3.20*

**If 'no' selected display question 3.21*

3.20 What potential risks are you aware of?

Answer ________________

**If protein Ready To Drink in question 3.1 is selected display question 3.21*

3.21 Why do you consume protein Ready-To-Drink?

Answer ________________

3.22 Have you personally experienced any problems or side effects from consuming protein Ready-To-Drink?

- o Yes
- o No

**If 'yes' selected display question 3.23*

**If 'no' selected display question 3.24*

3.23 What sort of side effects have you experienced?

Answer ________________

3.24 Are you aware of any potential risks associated with consuming protein Ready-To-Drink?

- o Yes
- o No

**If 'yes' selected display question 3.25*

**If 'no' selected display question 3.26*

3.25 What potential risks are you aware of?

Answer ________________

**If carbohydrate gel in question 3.1 is selected display question 3.26*

3.26 Why do you consume carbohydrate gels?

Answer ________________

3.27 Have you personally experienced any problems or side effects from consuming carbohydrate gels?

- Yes
- No

**If 'yes' selected display question 3.28*

**If 'no' selected display question 3.29*

3.28 What sort of side effects have you experienced?

Answer ________________

3.29 Are you aware of any potential risks associated with consuming carbohydrate gels?

- Yes
- No

**If 'yes' selected display question 3.30*

**If 'no' selected display question 3.31*

3.30 What potential risks are you aware of?

Answer ________________

**If carbohydrate powder in question 3.1 is selected display question 3.31*

3.31 Why do you consume carbohydrate powder?

Answer ________________

3.32 Have you personally experienced any problems or side effects from consuming carbohydrate powder?

- Yes
- No

**If 'yes' selected display question 3.33*

**If 'no' selected display question 3.34*

3.33 What sort of side effects have you experienced?

Answer ________________

3.34 Are you aware of any potential risks associated with consuming carbohydrate powder?

- o Yes
- o No

**If 'yes' selected display question 3.35*

**If 'no' selected display question 3.36*

3.35 What potential risks are you aware of?

Answer ________________

**If branch chain amino acid powder in question 3.1 is selected display question 3.36*

3.36 Why do you consume Branch Chain Amino Acids (BCAA)?

Answer ________________

3.37 Have you personally experienced any problems or side effects from consuming Branch Chain Amino Acids (BCAA)?

- o Yes
- o No

**If 'yes' selected display question 3.38*

**If 'no' selected display question 3.39*

3.38 What sort of side effects have you experienced?

Answer ________________

3.39 Are you aware of any potential risks associated with consuming Branch Chain Amino Acids (BCAA)?

- o Yes
- o No

**If 'yes' selected display question 3.40*

**If 'no' selected display question 3.41*

3.40 What potential risks are you aware of?

Answer ________________

**If creatine powder in question 3.1 is selected display question 3.41*

3.41 Why do you consume creatine?

Answer ________________

3.42 Have you personally experienced any problems or side effects from consuming creatine?

- o Yes
- o No

**If 'yes' selected display question 3.43*

**If 'no' selected display question 3.44*

3.43 What sort of side effects have you experienced?

Answer ________________

3.44 Are you aware of any potential risks associated with creatine?

- o Yes
- o No

**If 'yes' selected display question 3.46*

**If 'no' selected display question 3.47*

3.45 What potential risks are you aware of?

Answer ________________

**If glutamine powder in question 3.1 is selected display question 3.46*

3.46 Why do you consume glutamine?

Answer ________________

3.47 Have you personally experienced any problems or side effects from consuming glutamine?

- o Yes
- o No

**If 'yes' selected display question 3.48*

**If 'no' selected display question 3.49*

3.48 What sort of side effects have you experienced?

Answer ________________

3.49 Are you aware of any potential risks associated with consuming glutamine?

- o Yes
- o No

**If 'yes' selected display question 3.50*

**If 'no' selected display question 3.51*

3.50 What potential risks are you aware of?

Answer ________________

**If beta alanine powder in question 3.1 is selected display question 3.51*

3.51 Why do you consume beta alanine?

Answer ________________

3.52 Have you personally experienced any problems or side effects from consuming beta alanine?

- o Yes
- o No

**If 'yes' selected display question 3.53*

**If 'no' selected display question 3.54*

3.53 What sort of side effects have you experienced?

Answer ________________

3.54 Are you aware of any potential risks associated with consuming beta alanine?

- o Yes
- o No

**If 'yes' selected display question 3.55*

**If 'no' selected display question 3.56*

3.55 What potential risks are you aware of?

Answer ________________

**If L-Carnitine powder in question 3.1 is selected display question 3.42*

3.56 Why do you consume L-carnitine?

Answer ________________

3.57 Have you personally experienced any problems or side effects from consuming L-carnitine?

- o Yes
- o No

**If 'yes' selected display question 3.58*

**If 'no' selected display question 3.59*

3.58 What sort of side effects have you experienced?

Answer ________________

3.59 Are you aware of any potential risks associated with consuming L-carnitine?

- o Yes
- o No

**If 'yes' selected display question 3.60*

**If 'no' selected display question 3.61*

3.60 What potential risks are you aware of?

Answer ________________

**If pre-workout in question 3.1 is selected display question 3.61*

3.61 Why do you consume pre-workout?

Answer ________________

3.62 Have you personally experienced any problems or side effects from consuming pre-workout?

- o Yes

- No

**If 'yes' selected display question 3.63*

**If 'no' selected display question 3.64*

3.63 What sort of side effects have you experienced?

Answer ________________

3.64 Are you aware of any potential risks associated with consuming pre-workout?

- Yes
- No

**If 'yes' selected display question 3.65*

**If 'no' selected display question 3.66*

3.65 What potential risks are you aware of?

Answer ________________

**If other in question 3.1 is selected display question 3.66*

3.66 Why do you consume other formulated sports foods?

Answer ________________

3.67 Have you personally experienced any problems or side effects from consuming other formulated sports foods?

- Yes
- No

**If 'yes' selected display question 3.68*

**If 'no' selected display question 3.69*

3.68 What sort of side effects have you experienced?

Answer ________________

3.69 Are you aware of any potential risks associated with consuming other formulated sports foods?

- Yes

- o No

**If 'yes' selected display question 3.70*

**If 'no' selected display question 4.1*

3.70 What potential risks are you aware of?

Answer ________________

Page break --

Section 4: Physical activity questions

The following questions will be about exercise and physical activity participation.

This includes recreation activities, exercise in any form, or sport (matches and training for matches), activities you do at work, as part of your house and yard work, to get from place to place, and in your spare time.

Page break --

4.1 Have you participated in a sport/exercise/physical activity within the past 3 months?

- o Yes
- o No

**If answered 'yes' go to question 4.2*
**If answered 'no' go to end of block*

Page break --

4.2 Exercise type What sort of **sports/exercise/physical activity** have you participated in within the past 3 months? *(please tick all that apply)*

- ☐ Court ball sports (basketball, netball, tennis, badminton, table tennis)
- ☐ Field ball sports (football, hockey, soccer, rugby)
- ☐ Cycling
- ☐ Gym not elsewhere classified
- ☐ Weight training (body building, weightlifting, powerlifting, strength training)
- ☐ Martial arts
- ☐ Running/jogging
- ☐ Walking
- ☐ Water sports (rowing, surfing, swimming, water polo)
- ☐ Other (dancing, golf, horse riding, motor sports, yoga, Pilates)
- ☐ Active transport (commuting to and from work or study)
- ☐ Housework (cleaning, gardening)
- ☐ My work is physically demanding

Other (please specify)

Page break --

4.3 Exercise frequency The **sport/exercise/physical activity** you stated that you have participated within the past 3 months, how regularly do you do this?

	Very frequently (Multiple times per day, everyday, 5-6 times per week)	Frequently (2-3 times per week)	Once per week	Infrequently (Once per month ,2-3 times per month	Very infrequently (1-2 times per 3 months)
Court ball sports (basketball, netball, tennis, badminton, table tennis)	○	○	○	○	○
Field ball sports (football, hockey, soccer, rugby)	○	○	○	○	○
Cycling	○	○	○	○	○
Gym not elsewhere classified	○	○	○	○	○
Weight training (body building, weightlifting, powerlifting, strength training)	○	○	○	○	○
Martial arts	○	○	○	○	○
Running/jogging	○	○	○	○	○
Walking	○	○	○	○	○
Water sports (rowing, surfing, swimming, water polo)	○	○	○	○	○
Other (dancing, golf, horse riding, motor sports, yoga, Pilates)	○	○	○	○	○

Active transport (commuting to and from work or study)	○	○	○	○	○
Housework (cleaning, gardening)	○	○	○	○	○
My work is physically demanding	○	○	○	○	○
Other (please specify)	○	○	○	○	○

4.4 Exercise duration In the previous question you stated the frequency of participation in a **sport/exercise/physical activity.** Each time you complete a session, **how long** do you usually do this activity for?

	30 minutes or less per session	30 minutes up to 1 hour per session	1 up to 2 hours per session	2 up to 3 hours per session	3 hours or more per session
Court ball sports (basketball, netball, tennis, badminton, table tennis)	○	○	○	○	○
Field ball sports (football, hockey, soccer, rugby)	○	○	○	○	○
Cycling	○	○	○	○	○
Gym not elsewhere classified	○	○	○	○	○
Weight training (body building, weightlifting, powerlifting, strength training)	○	○	○	○	○
Martial arts	○	○	○	○	○
Running/jogging	○	○	○	○	○
Walking	○	○	○	○	○
Water sports (rowing, surfing, swimming, water polo)	○	○	○	○	○
Other (dancing, golf, horse riding, motor sports, yoga, Pilates)	○	○	○	○	○
Active transport (commuting to and from work or study)	○	○	○	○	○

Housework (cleaning, gardening)	○	○	○	○	○
My work is physically demanding	○	○	○	○	○
Other (please specify)	○	○	○	○	○

4.5 Is this the amount of sport/exercise/physical activity you would normally participate in?

- More than I would usually do
- About the same as I would usually do
- Less than I would usually do

Page break ---

Section 5: Packaging and personal recommendations

5.1 When you purchased formulated sports foods within the past 3 months, where did you purchase these from? *(Please tick all that apply)*

- Supermarket
- Health food store
- Supplement store
- Gym
- Personal trainer/coach
- Pharmacy or chemist
- Internet websites
- Other (please specify)

**If options 1-7 selected go to question 5.3*

Page break ---

5.2 How did you determine that formulated sports foods were right for you? (Please complete only those that apply)

- Friends
- Family member
- Peer groups (sports teammates/gym friendship group)
- Personal trainer/coach
- Gym receptionist
- Social media: profile advertising or influencer
- Internet or website advertising
- Television, newspaper, or magazine advertising

- Store-based retail employee
- Packaging claims or other statements
- Other (please specify)

**If options 1-12 selected only go to question 5.5*

Page break --

The following questions will be about **packaging and packaging attributes.**
These include:

- Written packaging information e.g., claims, marketing statements

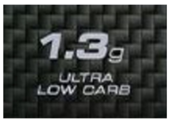

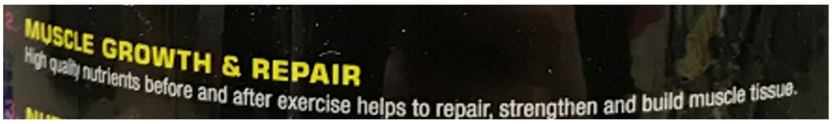

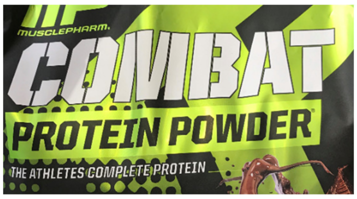

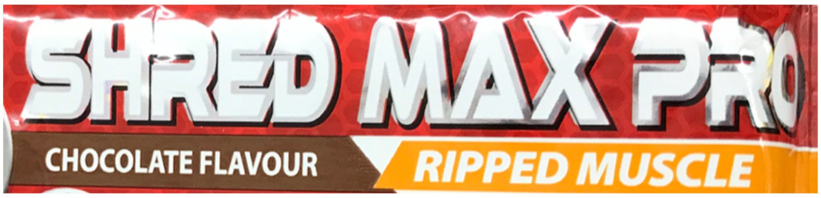

- Nutrition information e.g., nutrition information panel, ingredients list

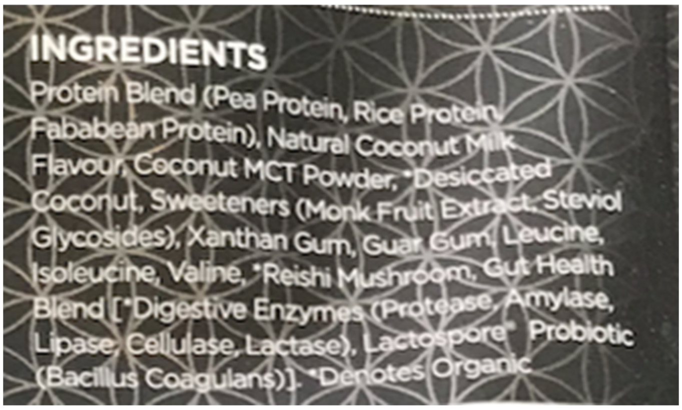

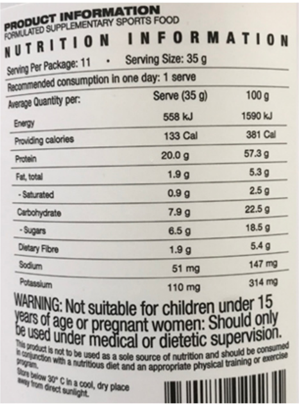

- Warning labels and advisory statements

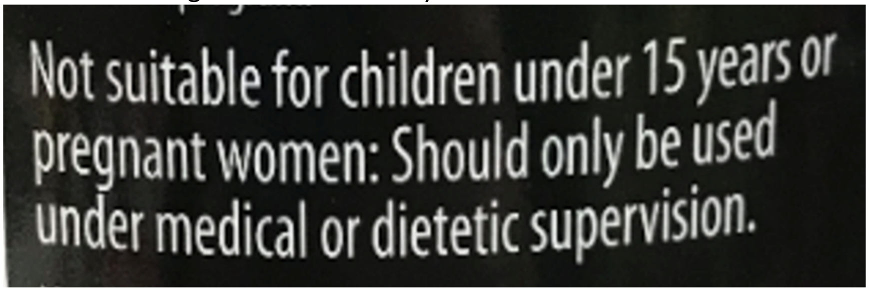

- Imagery displayed on the packaging

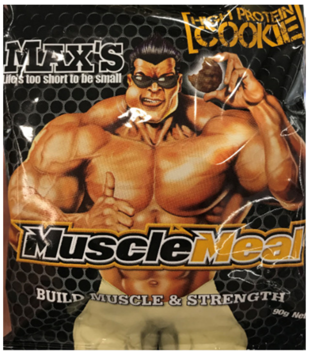

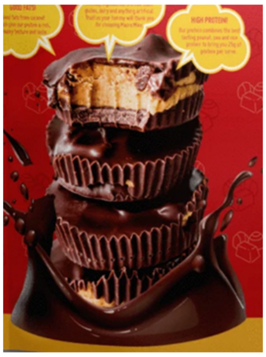

- Packaging colour

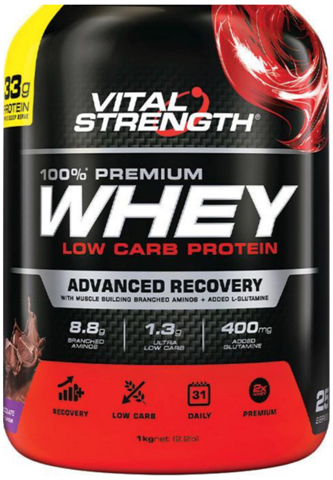

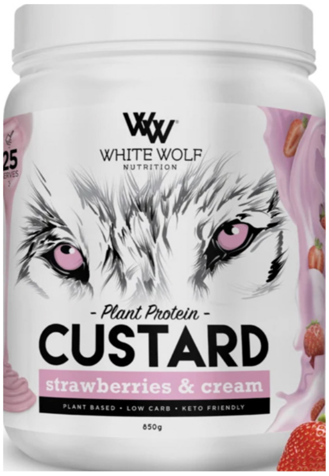

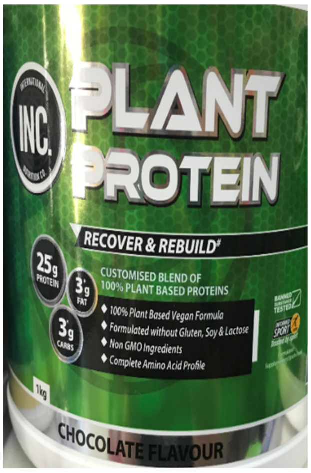

Page break --

5.3 When selecting **formulated sports foods**, which **packaging and packaging attributes** source/sources do you use to select products?
(Please select all that apply)

☐ **Written packaging information e.g., claims, marketing statements**

☐ **Nutrition information e.g., nutrition information panel, ingredients list**

☐ **Warning labels and advisory statements**

☐ **Imagery displayed on the packaging**

☐ **Packaging colour**

5.4 When you use **written/nutrition/warning information** to select these foods, what do you use to help your purchase decision?

Answer ________________

5.5 When you use **packaging imagery and/or colour** to select these foods, what do you use to help your purchase decision?

Answer ________________

5.6 how much of the information on the labels do you take notice of/read in helping you to make a purchasing decision?

- o All of the packaging information/label
- o Most of the packaging information/label
- o Some of the packaging information/label
- o None of the packaging information/label

5.7 Do you think the amount of information on the labels of formulated Sports Foods is?

- o Too much
- o About right
- o Too little

Page break --

***If you would like to be part of the incentive prize lottery, be contacted with the results of the study or if you would like to be contacted for future research, please select the opt in buttons below for each.**

- o **I would like to be part of the incentive prize draw**
- o **I would like to receive the results of the study**
- o **I would like to be contacted to be part of future studies examining Sports food packaging attributes**

Page break --

*** We thank you for your time spent taking this survey.**

Your responses have been recorded.

Press the SUBMIT button to lodge your responses.

Please note that once the submit button has been pressed you cannot withdraw your responses.
